# DIABETES MELLITUS

**DOI:** 10.1093/geroni/igz038.2129

**Published:** 2019-11-08

**Authors:** Steven J Prior

**Affiliations:** University of Maryland, Department of Kinesiology, College Park, Maryland, United States

## Abstract

Nearly three-fourths of adults over 65 year of age are affected by impaired glucose tolerance or type 2 diabetes. Both aging and inactivity contribute to the numerous skeletal muscle changes that occur with insulin resistance and type 2 diabetes. These changes include reduced capillarization that can impaired glucose uptake and substrate delivery, resulting in metabolic abnormalities and metabolic inflexibility. These changes may ultimately contribute to reduced delivery of oxygen, nutrients, and hormones to the muscles leading to impairments in metabolism, muscle mass, and function. We will discuss current research on the role of vascular impairments and reduced skeletal muscle capillarization in the development of impaired muscle metabolism, fitness and function. Finally, we will discuss how exercise training may reverse these declines.

